# Saffold Cardiovirus in Children with Acute Gastroenteritis, Beijing, China

**DOI:** 10.3201/eid1509.081531

**Published:** 2009-09

**Authors:** Lili Ren, Richard Gonzalez, Yan Xiao, Xiwei Xu, Lan Chen, Guy Vernet, Gláucia Paranhos-Baccalà, Qi Jin, Jianwei Wang

**Affiliations:** State Key Laboratory for Molecular Virology and Genetic Engineering, Beijing, People’s Republic of China (L. Ren, Q. Jin, J. Wang); Institute of Pathogen Biology, Beijing (L. Ren, R. Gonzalez, Y. Xiao, L. Chen, Q. Jin, J. Wang); Fondation Mérieux, Lyon, France (R. Gonzalez, G. Vernet, G. Paranhos-Baccalà); Beijing Children’s Hospital, Beijing (X. Xu)

**Keywords:** Saffold cardiovirus, children, acute gastroenteritis, viruses, Beijing, China, dispatch

## Abstract

To understand Saffold cardiovirus (SAFV) distribution, prevalence, and clinical relevance in China, we retrospectively studied SAFV in children with acute gastroenteritis and found SAFV in 12 (3.2%) of 373. Sequence homology of virus protein 1 genes suggested these strains belong to the SAFV-1 sublineage. SAFVs were found in samples positive for other diarrhea-causing viruses.

Recently, a new virus, provisionally named Saffold virus (SAFV), was recovered in the United States from a fecal sample from an 8-month-old girl with fever of unknown origin (*1*). The newly identified virus was classified under the genus *Cardiovirus,* family *Picornaviradae.* The 2 known species of the genus *Cardiovirus, encephalomyocarditis viruses* and *Theiler viruses* (*2*), are known to be pathogenic in several animal species and in humans (*3*–*6*). SAFV is genetically related to *Theiler viruses* and is believed to constitute a novel cardiovirus species (*1*,*7*). SAFV has been detected in children with enteric or respiratory tract infections in the United States, Canada, Brazil, Germany, Pakistan, and Afghanistan (*1*,*8*–*11*). However, the worldwide distribution of SAFV and its clinical significance remain unclear. To understand SAFV distribution, prevalence, and clinical relevance in the People’s Republic of China, we conducted a retrospective study by screening for SAFV in children with acute gastroenteritis.

## The Study

From March 2006 through November 2007, fecal samples were collected from 373 pediatric outpatients at Beijing Children’s Hospital in a prospective study on viral etiology of diarrhea. The ages of patients ranged from 1 month to 13 years (mean age 11.7 months, median age 9.0 months). Gastroenteritis was defined as acute watery diarrhea accompanied by other clinical signs and symptoms such as fever, nausea, and vomiting. No patient had any apparent clinical respiratory signs or symptoms.

We diluted these previously collected fecal specimens to 10% (wt/vol) with phosphate-buffered saline (pH 7.2) and removed cellular debris by centrifugation (2,500× *g* for 5 min). Virus nucleic acids were extracted by using the NucliSens miniMAG platform according to the manufacturer’s instructions (bioMérieux, Marcy l’Etoile, France). SAFV RNA was detected in the samples by nested reverse transcription–PCR (RT-PCR) that used primers targeting the 5′ untranslated region (UTR), which generated a 540-bp amplicon (*9*). Primers cardioVP1-1F/4R and cardioVP1-2F/3R, which span the virus protein 1 (VP1) gene, were used for a nested RT-PCR to amplify the VP1 gene (about 910 bp) as previously described (*10*). Because VP1 genes of 2 SAFV-positive samples could not be amplified in this way, a newly designed primer pair (cardioVP1Fn: TCAGAATGCCAATCTCCCCAAC and cardioVP1Rn: AAAGGTCCACCCGATACATTGA) was used in combination with cardioVP1-2F/3R to amplify the VP1 gene based on the sequences obtained from our positive samples. Conditions for first- and second-round PCR were 94°C for 3 min, followed by 40 cycles of 94°C for 30 sec, 48°C for 30 sec, and 72°C for 90 sec, and a final 10-min cycle at 72°C. All positive PCR amplicons were verified by sequencing after being cloned into the pMD18T vector (Takara Bio Inc., Dalian, China). Three positive clones were randomly selected for parallel sequencing. Each sample was screened by PCR for enteric adenovirus, astrovirus, noroviruses, sapovirus, and human bocavirus by using PCR (*12*,*13*) and for group A rotaviruses by using the Rotavirus ELISA Diagnostic Kit (Lanzhou Institute for Biological Products, Lanzhou, China). To characterize the nucleotide sequences obtained from this study, we analyzed the 5′ UTR and VP1 genes of all SAFV isolates to determine the extent of homology among the genes and those documented in the GenBank database by using MEGA software (*14*).

SAFV RNA was detected in 12 (3.2%) of 373 fecal specimens by using RT-PCR with primers targeting the 5′ UTR gene. Of these 12 positive specimens, 5 were collected from boys and 7 from girls. The ages of SAFV-positive patients ranged from 1 month to 3 years (mean age 12.3 months, median age 9.5 months). This age distribution is similar to that reported by Chiu et al., who detected SAFV mainly in younger children (*10*).

Co-infections with other viruses, including rotavirus (7/11) and norovirus (5/11), were detected in 11/12 SAFV-positive specimens (Table). The prevalence of rotavirus and norovirus infection in this sample pool was 59.5% (222/373) and 12.1% (45/373), respectively. SAFV-positive samples were found only in the last month of the 18-month study period, November 2007.

**Table:** Fecal samples positive for Saffold cardiovirus, Beijing, People’s Republic of China, March 2006–November 2007*

Sample no.	Patient sex/age, mo	Codetected virus
GL311	F/10	Rotavirus
GL317	F/20	None
GL328	M/24	Rotavirus
GL341	F/36	Rotavirus
GL352	F/6	Norovirus
GL361	F/11	Norovirus
GL362	M/1	Norovirus
GL365	M/10	Rotavirus
GL368	F/7	Rotavirus
GL371	F/9	Norovirus
GL376	M/8	Rotavirus
GL377	M/5	Rotavirus + norovirus

*All patients were from urban areas of Beijing and had acute gastroenteritis.

To assess the sequence variations of SAFV strains detected in this study, we analyzed an 825-bp cDNA fragment (GenBank accession nos. FJ464766–FJ464777) corresponding to the VP1 gene of the 12 SAFV strains. Previous studies have demonstrated the existence of 8 distinct phylogenetic sublineages of SAFV on the basis of the homology of P1 and VP1 genes (*9*,*11*). However, all strains identified in this study appeared to be the SAFV-1 sublineage (Figure), and they showed 99.2%–100% homology in nucleotide sequences and 98.5%–100% homology in the amino acid sequence of the VP1 gene. The identity of the VP1 amino acid sequences among these strains and the prototype SAFV (EF165067) was as high as 98.1%–98.9%. Multiple-alignment analysis showed that the amino acid sequence identity of VP1 among all available isolates varied from 62.3% to 100% (Appendix Table), indicating the global diversity of the SAFV strains from different geographic locations.

**Figure:**
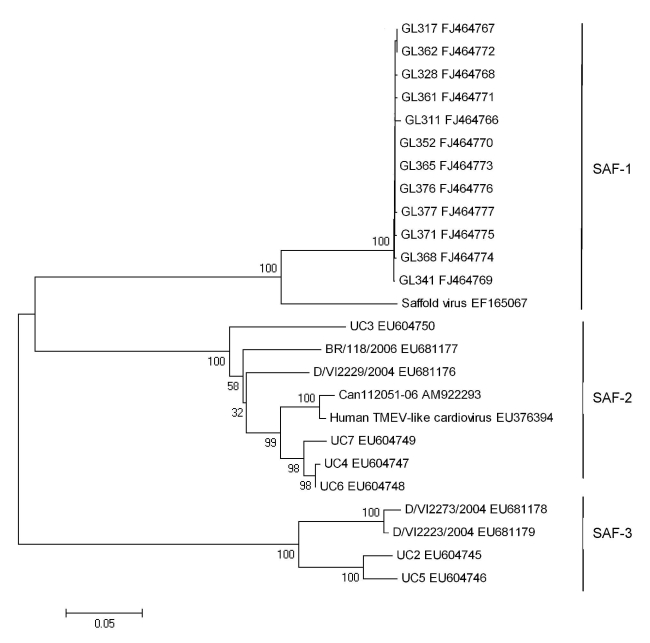
Phylogenetic analysis of nucleotide sequences of the virus protein 1 (VP1) gene of Saffold cardiovirus. The tree was constructed by using the Molecular Evolutionary Genetics Analysis (MEGA) software version and the neighbor-joining algorithm with kimura-2 parameters (*14*). The analysis included human Theiler murine encephalomyelitis virus (TMEV)–like cardiovirus. TMEV-like cardiovirus sequences (GenBank accession no. EU376394) and the previously reported SAFV sequences including the prototype SAFV, U2-U7, Can112051-06, BR/118/2006, D/VI2229/2004, D/VI2223/2004, and D/VI2273/2004 (GenBank accession nos. EF165067, NC009448, EU604745-EU604750, AM922293, and EU681176-EU681179) as references. Because the sequences of SAFV-4 to SAFV-8 are not available, they are not included in the phylogenetic tree. Each strain from this study is indicated by a specific identification code (GL) followed by the patient number (GL311, GL317, GL328, GL341, GL352, GL361, GL362, GL365, GL368, GL371, GL376, and GL377) and its GenBank accession number. Scale bar indicates nucleotide substitutions per site.

## Conclusions

This retrospective study showed that 12 (3.2%) of 373 children with acute gastroenteritis in Beijing were SAFV-positive, indicating the prevalence of recently characterized SAFV in China and providing evidence of global distribution of SAFV. Although SAFVs have been detected in samples collected from enteric and respiratory tracts, the clinical role for SAFV is still unclear. In this study, 11 of 12 SAFV-positive samples were co-infected with at least 1 known diarrhea-causing virus, such as rotavirus or norovirus. Therefore, we cannot conclusively state that SAFV is responsible for gastroenteritis (*8*–*11*).

That all SAFV-positive samples were collected in November 2007 suggests a possible seasonal outbreak. However, because of insufficient background information from outpatients, whether the rate of SAFV detection peaks in a single month or whether it indicates a seasonal outbreak is unclear. Further investigations are necessary. Nevertheless, our finding suggests a potential epidemic of SAFV during the cold season (*9*).

In the samples used for this study, we detected SAFV-1 only, no other sublineages (*9*,*11*). Although isolated 20 years ago in San Diego, California, USA, the SAFV-1 sequence was not published until 2007 because of the progress of molecular techniques for unknown genome cloning (*1*). The reason for this 20-year hiatus of SAFV-1 in the United States and the occurrence of the same genotype in China in 2007 is unclear. It can be attributed to a geographic variation of an SAFV epidemic in the world. Currently, 8 SAFV sublineages have been identified in different areas: SAFV-2 and SAFV-3 have been detected in North and South America (United States and Brazil) and Europe (*9**, **10*)*,* and SAFV-2 to SAFV-8 have been detected in South Asia (*11*)*.* SAFV-1 may be the dominant sublineage in circulation in Beijing and may have escaped detection until the current investigation. The dominant SAFV sublineages may be changing over time in a certain geographic area. An SAFV-1 endemic to the United States in the1980s may have been subsequently replaced by SAFV-2 and SAFV-3, whereas SAFV-1 has now become dominant in Beijing. Because the existence of these human Theiler-like viruses was unknown before 2007, comprehensive global investigations of the prevalence and diversity of SAFV, especially studies based on samples collected over the previous years, will be helpful in providing further insights into SAFV origin, sublineage, and distribution.

## Supplementary Material

Appendix Table Amino acid and nucleotide acid sequence identities of VP1 sequences between SAFV strains, Beijing, People's Republic of China, March 2006-November 2007*†
