# Supplementary material for: Saffold Cardiovirus in Children with Acute Gastroenteritis, Beijing, China
Source: Emerg Infect Dis. 2009 Sep;15(9):1509–11. doi: 10.3201/eid1509.081531 (PMC2819865; doi:10.3201/eid1509.081531)
Supplement: Appendix Table — Amino acid and nucleotide acid sequence identities of VP1 sequences between SAFV strains, Beijing, People's Republic of China, March 2006-November 2007*† [file 08-1531_appT-s1.pdf]

Publisher: CDC; Journal: Emerging Infectious Diseases
Article Type: Dispatch; Volume: 15; Issue: 9; Year: 2009; Article ID: 08-1531
DOI: 10.3201/eid1509.081531; TOC Head: Dispatch

Appendix Table. Amino acid and nucleotide acid sequence identities of VP1 sequences between SAFV strains, Beijing, People's Republic of China, March 2006–November 2007*†

Sublineage and strain	[1]	[2]	[3]	[4]	[5]	[6]	[7]	[8]	[9]	[10]	[11]	[12]	[13]	[14]	[15]	[16]	[17]	[18]	[19]	[20]	[21]	[22]	[23]	[24]	[25]
SAFV-1																									
[1] GL311																									
[2] GL317	99.2/ 99.3																								
[3] GL328	98.9/ 99.3	99.6/ 99.7																							
[4] GL341	98.5/ 99.2	99.2/ 99.6	98.9/ 99.6																						
[5] GL352	99.2/ 99.5	100.0/ 99.8	99.6/ 99.8	99.2/ 99.7																					
[6] GL361	98.9/ 99.3	99.6/ 99.7	99.2/ 99.7	98.9/ 99.6	99.6/ 99.8																				
[7] GL362	99.2/ 99.3	100.0/ 100.0	99.6/ 99.7	99.2/ 99.6	100.0/ 99.8	99.6/ 99.7																			
[8] GL365	99.2/ 99.5	100.0/ 99.8	99.6/ 99.8	99.2/ 99.7	100.0/ 100.0	99.6/ 99.8	100.0/ 99.8																		
[9] GL368	99.2/ 99.3	100.0/ 99.7	99.6/ 99.7	99.2/ 99.6	100.0/ 99.8	99.6/ 99.7	100.0/ 99.7	100.0/ 99.8																	
[10] GL371	99.2/ 99.3	100.0/ 99.7	99.6/ 99.7	99.2/ 99.7	100.0/ 99.8	99.6/ 99.7	100.0/ 99.7	100.0/ 99.8	100.0/ 99.7																
[11] GL376	99.2/ 99.5	100.0/ 99.8	99.6/ 99.8	99.2/ 99.7	100.0/ 100.0	99.6/ 99.8	100.0/ 99.8	100.0/ 100	100.0/ 99.8	100.0/ 99.8															
[12] GL377	99.2/ 99.3	100.0/ 99.7	99.6/ 99.7	99.2/ 99.6	100.0/ 99.8	99.6/ 99.7	100.0/ 99.7	100.0/ 99.8	100.0/ 99.7	100.0/ 99.7	100.0/ 99.8														
[13] Saffold_virus	98.1/ 86.3	98.9/ 86.6	98.5/ 86.6	98.1/ 86.6	98.9/ 86.7	98.5/ 86.6	98.9/ 86.6	98.9/ 86.7	98.9/ 86.6	98.9/ 86.6	98.9/ 86.7	98.9/ 86.7													
SAFV-2																									
[14] Can112051-06	74.1/ 66.8	74.9/ 67.0	74.5/ 66.9	74.1/ 66.9	74.9/ 67.0	74.5/ 66.9	74.9/ 67.0	74.9/ 67.0	74.9/ 67.0	74.9/ 66.9	74.9/ 67.0	74.9/ 67.0	75.6/ 67.3												
[15] D/VI2229/2004	73.0/ 66.2	73.8/ 66.7	73.4/ 66.5	73/ 66.8	73.8/ 66.7	73.4/ 66.5	73.8/ 66.7	73.8/ 66.7	73.8/ 66.7	73.8/ 66.7	73.8/ 66.7	73.8/ 66.7	74.5/ 65.4	95.9/ 88.8											
[16] BR/118/2006	74.5/ 67.3	75.2/ 67.5	74.9/ 67.4	74.5/ 67.4	75.2/ 67.5	74.9/ 67.4	75.2/ 67.5	75.2/ 67.5	75.2/ 67.5	75.2/ 67.4	75.2/ 67.5	75.2/ 67.5	76/ 67.4	97.7/ 90.5	95.9/ 89.7										
[17] HTCV_EU376394	74.1/ 67.1	74.9/ 67.4	74.5/ 67.3	74.1/ 67.3	74.9/ 67.4	74.5/ 67.3	74.9/ 67.4	74.9/ 67.4	74.9/ 67.4	74.9/ 67.3	74.9/ 67.4	74.9/ 67.4	75.6/ 67.6	99.2/ 98.6	96.6/ 89.7	98.5/ 90.7									
[18] UC3	72.7/ 66.5	73.4/ 66.8	73.0/ 66.7	72.7/ 66.7	73.4/ 66.8	73/ 66.7	73.4/ 66.8	73.4/ 66.8	73.4/ 66.8	73.4/ 66.7	73.4/ 66.8	73.4/ 66.8	74.1/ 66.3	96.3/ 87.9	94.8/ 85.8	95.9/ 87.7	97.0/ 88.1								
[19] UC4	73.8/ 67.4	74.5/ 67.6	74.1/ 67.5	73.8/ 67.5	74.5/ 67.6	74.1/ 67.5	74.5/ 67.6	74.5/ 67.6	74.5/ 67.6	74.5/ 67.5	74.5/ 67.6	74.5/ 67.6	75.2/ 67.1	98.5/ 94.7	96.6/ 90.4	98.1/ 90.3	99.2/ 95.4	97.0/ 89.6							
[20] UC6	74.1/ 67.6	74.9/ 67.9	74.5/ 67.7	74.1/ 67.7	74.9/ 67.9	74.5/ 67.7	74.9/ 67.9	74.9/ 67.9	74.9/ 67.9	74.9/ 67.7	74.9/ 67.9	74.9/ 67.9	75.6/ 67.4	98.8/ 94.7	97.0/ 90.4	98.5/ 90.3	99.6/ 95.4	97.4/ 89.6	99.6/ 99.7						
[21] UC7	73.8/ 67.3	74.5/ 67.5	74.1/ 67.4	73.8/ 67.4	74.5/ 67.5	74.1/ 67.4	74.5/ 67.5	74.5/ 67.5	74.5/ 67.5	74.5/ 67.4	74.5/ 67.5	74.5/ 67.5	75.2/ 66.9	98.5/ 93.4	96.6/ 89.2	98.1/ 89.2	99.2/ 93.8	97.0/ 89.0	99.2/ 97.6	99.6/ 97.6					
SAFV-3																									
[22] D/VI2273/2004	65.9/ 62.7	66.6/ 62.8	66.3/ 62.8	66.6/ 62.8	66.6/ 62.9	66.3/ 62.8	66.6/ 62.8	66.6/ 62.9	66.6/ 63.0	66.6/ 62.8	66.6/ 62.9	66.6/ 62.8	66.3/ 62.9	72.1/ 65.8	70.6/ 63.4	71.7/ 64.7	71.7/ 65.7	69.9/ 65.2	71.4/ 64.7	71.7/ 64.7	71.4/ 65.3				
[23] D/VI2223/2004	65.9/ 63.1	66.6/ 63.3	66.3/ 63.3	66.6/ 63.3	66.6/ 63.4	66.3/ 63.3	66.6/ 63.3	66.6/ 63.4	66.6/ 63.5	66.6/ 63.3	66.6/ 63.4	66.6/ 63.3	66.3/6 2.9	72.1/ 66.4	70.6/ 64.2	71.7/ 65.3	71.7/ 66.3	69.9/ 65.3	71.4/ 65.1	71.7/ 65.1	71.4/ 65.7	100.0/ 98.6			

[24] UC2	65.9/ 62.8	66.6/ 63.1	66.3/ 63.1	66.6/ 63.4	66.6/ 63.3	66.3/ 63.1	66.6/ 63.1	66.6/ 63.3	66.6/ 63.4	66.6/ 63.3	66.6/ 63.3	66.6/ 63.1	66.3/ 63.8	72.1/ 65.3	71.0/ 64.8	72.1/ 65.3	72.1/ 65.2	70.3/ 63.9	71.7/ 64.6	72.1/ 64.6	71.7/ 64.8	98.8/ 8 8.4	98.8/ 88.8	
[25] UC5	65.9/ 62.3	66.6/ 62.4	66.3/ 62.4	66.6/ 62.7	66.6/ 62.5	66.3/ 62.4	66.6/ 62.4	66.6/ 62.5	66.6/ 62.7	66.6/ 62.5	66.6/ 62.5	66.6/ 62.4	66.3/ 63.4	72.1/ 65.9	71.0/ 65.9	72.1/ 65.6	72.1/ 65.6	70.3/ 64.8	71.7/ 64.8	72.1/ 64.8	71.7/ 65.6	98.8/ 88.6	98.8/ 89	100.0/ 96.0

*VP1, virus protein 1; SAFV, Saffold cardiovirus; HTCV, human Theiler's murine encephalomyelitis virus–like cardiovirus.

†All results are based on the pairwise analysis of 25 currently available sequences of SAFV VP1 genes by using MEGA version 4.0 (*14*). Identification of these SAFV strains is shown in the legend of the Figure. The final dataset contained a total of 273 positions. The results showed the similarity in amino acids and nucleotide sequences between these positive samples and the VP1 of other SAFVs. Because the sequences of SAFV-4 to SAFV-8 are not available, they are not included in amino acid and nucleotide acid sequence identity analysis. Values are expressed as identity of amino acid sequences/identity of nucleotide sequences.
